# Progress Realized: Trends in HIV-1 Viral Load and CD4 Cell Count in a Tertiary-Care Center from 1999 through 2011

**DOI:** 10.1371/journal.pone.0056845

**Published:** 2013-02-20

**Authors:** Howard B. Gale, Manuel D. Rodriguez, Heather J. Hoffman, Debra A. Benator, Fred M. Gordin, Ann M. Labriola, Virginia L. Kan

**Affiliations:** 1 Infectious Diseases Section, Medical Service, Veterans Affairs Medical Center, Washington, D.C., United States of America; 2 Division of Infectious Diseases, The George Washington University, Washington, D.C., United States of America; 3 Department of Epidemiology and Biostatistics, The George Washington University, Washington, D.C., United States of America; University of Malaya, Malaysia

## Abstract

**Background:**

HIV-1 RNA and CD4 cell counts are important parameters for HIV care. The objective of this study was to assess the overall trends in HIV-1 viral load and CD4 cell counts within our clinic.

**Methods:**

Patients with at least one of each test performed by the Infectious Diseases Laboratory from 1999 through 2011 were included in this analysis. By adapting a novel statistical model, log_10_ HIV-1 RNA means were estimated by month, and log_10_-transformed HIV-1 RNA means were estimated by calendar year. Geometric means were calculated for CD4 cell counts by month and calendar year. Log_10_ HIV-1 RNA and CD4 cell count monthly means were also examined with polynomial regression.

**Results:**

There were 1,814 individuals with approximately 25,000 paired tests over the 13-year observation period. Based on each patient's final value of the year, the percentage of patients with viral loads below the lower limit of quantitation rose from 29% in 1999 to 72% in 2011, while the percentage with CD4 counts <200 cells/µL fell from 31% to 11%. On average annually, the mean HIV-1 RNA decreased by 86 copies/mL and the mean CD4 counts increased by 16 cells/µL. For the monthly means, the correlations (R^2^) from second-order polynomial regressions were 0.944 for log_10_ HIV-1 RNA and 0.840 for CD4 cell counts.

**Conclusions:**

Marked improvements in HIV-1 RNA suppression and CD4 cell counts were achieved in a large inner-city population from 1999 through 2011. This success demonstrates that sustained viral control with improved immunologic status can be a realistic goal for most individuals in clinical care.

## Introduction

Care for HIV-infected patients has changed dramatically over the last three decades [1] largely due to advances in antiretroviral therapies, which have allowed improvements in HIV-1 viral loads and CD4 cell counts. Because of these gains, the life expectancy of individuals diagnosed with HIV who are able to maintain fully suppressive antiretroviral regimens, now approaches those without infection [2]. However, inner-city and veteran populations with serious co-morbidities can present special challenges to achieving these gains. These comorbidities [3]–[6] which often include alcohol and substance abuse [7]–[9] and mental illness [10]–[12] can lead to concurrent disease manifestations and drug-drug interactions. Compared to those without infection, HIV-infected patients also have higher rates of poor treatment adherence due to lack of family/social support, adverse drug effects, complex drug regimens, psychological distress, and low patient self-efficacy [13],[14]. In addition, combination antiretroviral therapy regimens have been associated with many adverse side effects including metabolic changes and drug toxicities [15] as well as development of drug resistance [16], thus leading to virologic failure and poor clinical outcomes [17].

In order to respond to these complexities, our medical center's Infectious Diseases Clinic has provided HIV and primary care in a comprehensive model with an on-site, multidisciplinary team of nurses, physicians, social workers, pharmacists, and medical subspecialists. In this evaluation, we reviewed the HIV-1 viral loads and CD4 cell counts from 1999 through 2011 to determine the overall trends in viral load reduction and immune reconstitution across the entire spectrum of patients receiving HIV treatment in an inner-city setting. A novel statistical model was adapted to estimate the HIV-1 RNA values outside of the quantitative range.

## Methods

We retrospectively evaluated every HIV-1 RNA and paired CD4 cell count performed by the Infectious Diseases Laboratory for all patients tested for both parameters at least once from January 1999 through December 2011 at the Washington DC Veterans Affairs Medical Center. This evaluation included all HIV-infected persons who received care at the clinic without regard to whether the person was prescribed antiretroviral therapy. No charts were reviewed.

The Infectious Diseases Laboratory performs the clinical HIV-1 RNA and CD4 cell counts for our medical center. Written consent was not needed from patients as these tests were performed for clinical indications and not specifically for research purposes. As a clinical laboratory, we maintain databases of these test results. For purposes of the present analyses, we de-identified the datasets. Because we used de-identified, limited datasets, our medical center's IRB deemed this study to be exempt from the board's review since it would pose minimal risk for patients' privacy and data confidentiality.

Although our laboratory performed HIV-1 RNA testing prior to 1999, the lower limit of quantitation for that method was 500 copies/mL and it quantitated 1.5- to 4.5-fold lower than the two subsequent assays used in this report, Versant HIV-1 RNA 3.0 Assay (bDNA) (Siemens Healthcare Diagnostics Inc., Tarrytown, NY) and Abbott RealTime HIV-1 (Abbott Molecular Inc., Des Plaines, IL). These latter assays produced equivalent results in the quantitative range [18]. For CD4 cell count determinations, leukocyte counts were obtained from a hematology analyzer, and the percent lymphocytes and %CD4+ T-lymphocytes were analyzed by flow cytometry [19] using a FACSCalibur or FACSCanto II flow cytometer (BD Biosciences, San Jose, CA). The leukocyte count, percent lymphocytes and %CD4+ T-lymphocytes were then multiplied to calculate the CD4 cell count.

HIV-1 RNA data were transformed using base-10 logarithms. In order to minimize bias, two-limit Tobit, censored regression models (Supporting Information, [20]) were used to estimate the true mean log_10_ values by month and year: simple substitutions of the censored data with one-half of the lower limit of quantitation (LLOQ) or with the fill-in value method exhibit bias when the percentage of measurements <LLOQ reach 5–10% and 30%, respectively [21]. Because CD4 cell counts were positively skewed, these data were also transformed by using base-10 logarithms. Arithmetic means of the log_10_-transformed CD4 cell counts were calculated by month and year. These means were then back transformed to obtain the geometric means.

For both HIV-1 RNA and CD4 cell counts, polynomial regression models were fitted to their 156 monthly means to determine the trends. Residual plots were used to visually assess whether model assumptions were satisfied. In addition, the correlation properties of the residuals were analyzed using the identification stage of the Box-Jenkins approach to autoregressive integrated moving average (ARIMA) modeling [22]. Ljung-Box chi-square statistical tests were applied to test the null hypothesis that the set of autocorrelations was white noise; there was no information in the series to model, and no ARIMA model was needed for the series. The autocorrelations were checked in groups of six up to 24 lags. All statistical analyses were performed using SAS® software, Version 9.2 (SAS Institute, Cary, NC).

## Results

**Figure 1:**
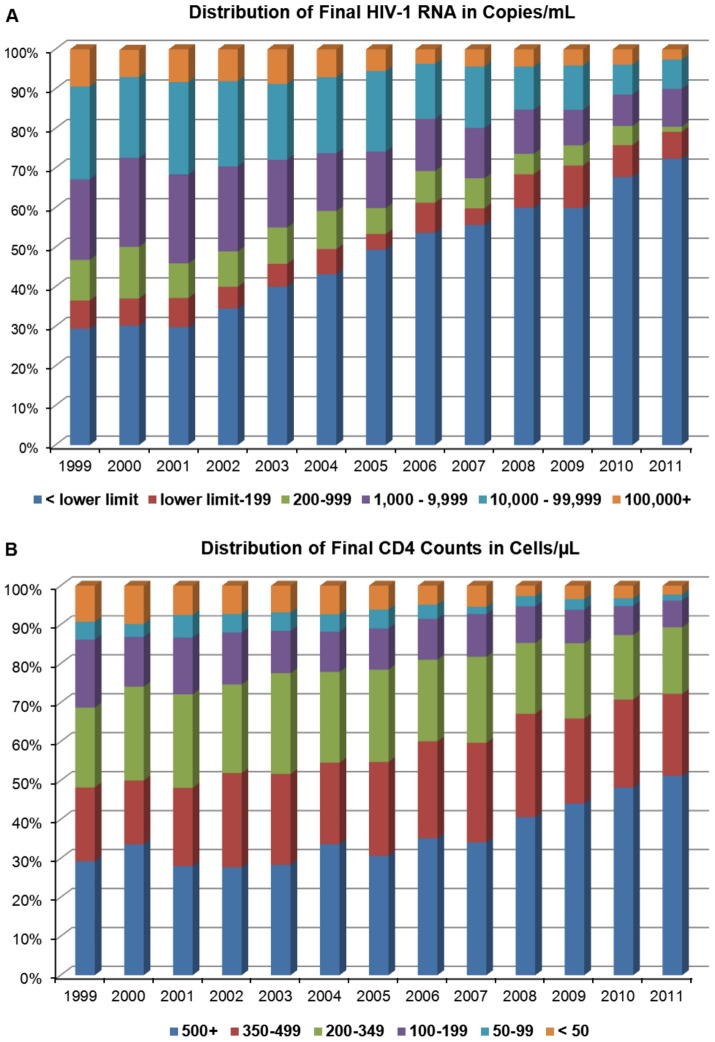
Distribution of final HIV-1 RNA and CD4 cell counts. (**A**) Final HIV-1 RNA and (**B**) final CD4 cell counts by calendar year for 1,814 unique patients tested for both parameters at least once from 1999 through 2011. The number of individuals each year ranged from 575 in 1999 to 854 in 2011. The HIV-1 RNA lower limit of quantitation was 50 copies/mL from 1-1-99 to 10-17-02, 75 copies/mL from 10-18-02 to 3-4-08 and 40 copies/mL from 3-5-08 to 12-31-11.

**Figure 2:**
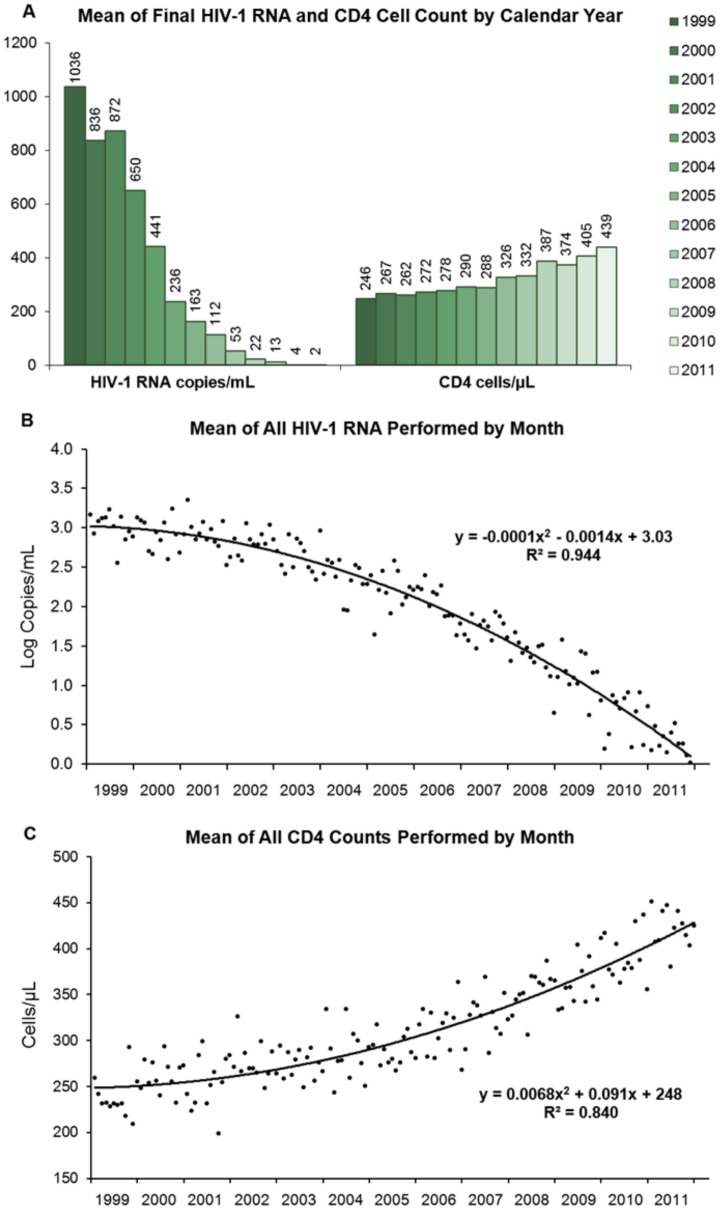
Annual and monthly means of HIV-1 RNA and CD4 cell counts. (**A**) Annual log_10_-transformed HIV-1 RNA means and CD4 cell count geometric means calculated from each patient's final values by calendar year. (**B**) Monthly log_10_ HIV-1 RNA means and (**C**) CD4 cell count geometric means calculated from all testing in a given month which represented 21–24% of that year's number of patients (575 persons in 1999 to 854 in 2011). Two-limit Tobit, censored regression models were used to estimate the true mean log_10_ HIV-1 RNA values by month and year. CD4 cell counts of 0 were assigned the value of 1 cell/µL.

For 1,814 unique individuals, ranging from 575 persons in 1999 to 854 persons in 2011, there were a total of 25,678 HIV-1 RNA and 24,992 CD4 cell counts performed. As seen in Figure 1A, based on each patient's final value of the year, the percentage of patients with viral loads below the lower limit of quantitation rose from 29% in 1999 to 72% in 2011 (Table S1), while the percentage with CD4 counts <200 cells/µL fell from 31% to 11% during that same period of time (Figure 1B and Table S2). On average annually, the mean HIV-1 RNA decreased by 86 copies/mL and the mean CD4 counts increased by 16 cells/µL (Figure 2A). For the monthly means, the correlations (R^2^) were 0.944 for log_10_ HIV-1 RNA (Figure 2B) and 0.840 for CD4 cell counts (Figure 2C).

## Discussion

This evaluation demonstrates the profound impact on the course of HIV that can be achieved in a broadly diverse inner-city clinic. From the beginning of our observation period in 1999, HIV-1 viral loads improved remarkably such that 72% of our patients measured below the lower limit of quantitation by the end of 2011. Importantly, CD4 cell counts also improved such that 89% of our patients had ≥200 cells/µL by the end of 2011, above the generally recognized threshold for opportunistic infections [23]. Furthermore, our data includes information on persons who were not prescribed antiretroviral treatment based on CD4 cell count or patient-centered issues. Therefore, despite inclusion of these persons in the analysis, the viral burden of the overall clinic population markedly decreased over the 13 years of this evaluation. These gains are particularly meaningful given patients' significant barriers to success [3]–[17]. In our clinic, more than half of our veterans have major medical, mental health or substance abuse issues.

Our study has the advantage of using a complete data set from a single inner-city setting to provide comprehensive trends for HIV-1 viral loads and CD4 cell counts within the same observation period and include data through 2011. Earlier analyses focused solely on viral loads [24]–[26] or CD4 cell counts [27],[28]. A recent publication [29] did analyze HIV-1viral loads and CD4 cell count trends for multiple U.S. sites through 2008 and examined median CD4 cell counts only at death. Our data on viral loads can be compared to the information on antiretroviral therapy in the Medical Monitoring Project in United States during 2008–2010 [30]. When using viral suppression as defined by a viral load of <200 copies/mL, 79% of our patients met this criterion, which was comparable to the estimated 77% of persons with HIV suppression in the Medical Monitoring study. However, this study did not include CD4 count monitoring.

The advantages of multidisciplinary teams providing medical and psychosocial support have been documented [14],[31]. Integrated care, pharmacists' assistance, co-location of mental illness services, and psychosocial well-being have been important in retention to care [32]–[35], as more HIV-infected veterans reported a lower quality of life compared to non-infected veterans [36]. Our findings highlight that incorporating these approaches to HIV care, can help patients attain the benefits of highly effective antiretroviral treatment. Another valuable tool for us has been the robust electronic medical record of the Veterans Affairs healthcare system that allows the quick review of medication renewals in order to uncover adherence issues during clinic visits.

In conclusion, our clinic patients attained striking improvements in HIV-1 RNA suppression and CD4 cell counts from 1999 through 2011, demonstrating that these goals can be realistically achieved for most individuals in clinical care.

## Supporting Information

Model S1 **Tobit model description.**
(DOCX)

Table S1 **Distribution of the final HIV-1 RNA for individual patients by calendar year.**
(DOC)

Table S2 **Distribution of the final CD4 cell counts for individual patients by calendar year.**
(DOC)
