# Supplementary material for: Progress Realized: Trends in HIV-1 Viral Load and CD4 Cell Count in a Tertiary-Care Center from 1999 through 2011
Source: PLoS One. 2013 Feb 20;8(2):e56845. doi: 10.1371/journal.pone.0056845 (PMC3577700; doi:10.1371/journal.pone.0056845)
Supplement: Model S1 — Tobit model description. (DOCX) [file pone.0056845.s001.docx]

**Tobit Model**

December 2012

Heather J. Hoffman, PhD

Associate Professor of Biostatistics

The George Washington University School of Public Health and Health Services

[hhoffman@gwu.edu](mailto:hhoffman@gwu.edu)

The structural equation in the Tobit model is:

$$y_{i}^{*}=X_{i}\beta+\varepsilon_{i}$$

where $\varepsilon_{i}\sim N\left( 0, \sigma^{2} \right)$ and $y_{i}^{*}$is a latent variable that is observed for values greater than $\tau$ and censored otherwise. The observed $y$ is defined by the following measurement equation:

$$y_{i}=\left\{ \begin{matrix} y^{*} & \text{if} y^{*}>\tau\\ \tau& \text{if} y^{*}\leq\tau\end{matrix} \right.$$

The likelihood function for the censored normal distribution is:

$$L=\prod_{i=1}^{N} \left[ \frac{1}{\sigma}\varphi\left( \frac{y-X_{i}\beta}{\sigma} \right) \right]^{I\left( y_{i} \right)}\left[ 1-\Phi\left( \frac{X_{i}\beta-\tau}{\sigma} \right) \right]^{1-I\left( y_{i} \right)}$$

where $\tau$ is the censoring point and

$$I\left( y_{i} \right)=\left\{ \begin{matrix} 0 & \text{if} y_{i}=\tau\\ 1 & \text{if} y_{i}\neq\tau\end{matrix} \right.$$

The overall likelihood is made up of two parts. The first part corresponds to the classical regression for the uncensored observations, while the second part corresponds to the relevant probabilities that an observation is censored.
