# Supplementary material for: Progress Realized: Trends in HIV-1 Viral Load and CD4 Cell Count in a Tertiary-Care Center from 1999 through 2011
Source: PLoS One. 2013 Feb 20;8(2):e56845. doi: 10.1371/journal.pone.0056845 (PMC3577700; doi:10.1371/journal.pone.0056845)
Supplement: Table S1 — Distribution of the final HIV-1 RNA for individual patients by calendar year. (DOC) [file pone.0056845.s002.doc]

**TABLE S1.** Distribution of the final HIV-1 RNA for individual patients by calendar year.

|  | **Percentage of HIV-1 RNA** | | | | | | | | | | | | |
| --- | --- | --- | --- | --- | --- | --- | --- | --- | --- | --- | --- | --- | --- |
| **Copies/mL** | **1999** | **2000** | **2001** | **2002** | **2003** | **2004** | **2005** | **2006** | **2007** | **2008** | **2009** | **2010** | **2011** |
| <LLOQ ^a^ | 29 | 30 | 30 | 34 | 40 | 43 | 49 | 54 | 56 | 60 | 60 | 68 | 72 |
| LLOQ–199 | 7 | 7 | 7 | 5 | 6 | 6 | 4 | 8 | 4 | 8 | 11 | 8 | 7 |
| 200–999 | 10 | 13 | 9 | 9 | 9 | 10 | 7 | 8 | 8 | 5 | 5 | 5 | 1 |
| 1,000–9,999 | 20 | 23 | 22 | 22 | 17 | 15 | 14 | 13 | 13 | 11 | 9 | 8 | 10 |
| 10,000–99,999 | 23 | 20 | 23 | 22 | 19 | 19 | 20 | 14 | 16 | 11 | 11 | 8 | 7 |
| >100,000 | 9 | 7 | 8 | 8 | 9 | 7 | 6 | 4 | 4 | 4 | 4 | 4 | 3 |

^a^ Lower limit of quantitation (LLOQ) was 50 copies/mL from 1-1-99 to 10-17-02, 75 copies/mL from 10-18-02

to 3-4-08, and 40 copies/mL from 3-5-08 to 12-31-11. A total of 1,814 unique patients had at least one test

performed during this period, ranging from 575 persons in 1999 to 854 in 2011.
