# Supplementary material for: Progress Realized: Trends in HIV-1 Viral Load and CD4 Cell Count in a Tertiary-Care Center from 1999 through 2011
Source: PLoS One. 2013 Feb 20;8(2):e56845. doi: 10.1371/journal.pone.0056845 (PMC3577700; doi:10.1371/journal.pone.0056845)
Supplement: Table S2 — Distribution of the final CD4 cell counts for individual patients by calendar year. (DOC) [file pone.0056845.s003.doc]

**TABLE S2.** Distribution of the final CD4 cell counts for individual patients by calendar year.

|  | **Percentage of CD4 Counts** | | | | | | | | | | | | |
| --- | --- | --- | --- | --- | --- | --- | --- | --- | --- | --- | --- | --- | --- |
| **Cells/µL** | **1999** | **2000** | **2001** | **2002** | **2003** | **2004** | **2005** | **2006** | **2007** | **2008** | **2009** | **2010** | **2011** |
| <50 | 9 | 10 | 8 | 7 | 7 | 7 | 6 | 5 | 5 | 3 | 3 | 3 | 2 |
| 50–99 | 5 | 3 | 6 | 5 | 5 | 4 | 5 | 4 | 2 | 3 | 3 | 2 | 2 |
| 100–199 | 17 | 13 | 15 | 13 | 11 | 10 | 11 | 10 | 11 | 9 | 9 | 7 | 7 |
| 200–349 | 21 | 24 | 24 | 23 | 26 | 23 | 24 | 21 | 22 | 18 | 19 | 17 | 17 |
| 350–499 | 19 | 16 | 20 | 24 | 23 | 21 | 24 | 25 | 26 | 27 | 22 | 23 | 21 |
| >500 | 29 | 34 | 28 | 28 | 28 | 34 | 31 | 35 | 34 | 41 | 44 | 48 | 51 |

A total of 1,814 unique patients had at least one test performed during this period, ranging from 575

persons in 1999 to 854 in 2011.
